# Exploring the prevalence and clinical impact of carotid plaque burden by Doppler ultrasound in lung cancer screening participants with limited coronary artery calcification

**DOI:** 10.1007/s00330-026-12390-1

**Published:** 2026-02-24

**Authors:** Rebecca Mura, Roberta Eufrasia Ledda, Luca Moderato, Ludovica Leo, Pasquale Favia, Carlotta Zilioli, Antonella Priore, Francesca Lucia Maffucci, Vita Ida Gallone, Camilla Roberti, Silvia Schirò, Mario Silva, Nicola Sverzellati, Gianluca Milanese

**Affiliations:** 1 Department of Medicine and Surgery (diMeC), University of Parma, Parma, Italy; 2 University Hospital of Parma, Parma, Italy; 3 Cardiology Unit, OSPEDALE OGLIO PO, Casalmaggiore, Italy

**Keywords:** Atherosclerosis, Carotid Doppler ultrasound, Lung cancer screening

## Abstract

**Objectives:**

This study aimed to evaluate the frequency of carotid plaques detected by carotid Doppler ultrasound (CDU) and their potential contribution to therapeutic recommendations, among participants in a lung cancer screening (LCS) programme—notably in those with absent or limited coronary artery calcification (CAC).

**Materials and methods:**

This prospective study included 250 individuals from the “PEOPLHE” LCS-trial who were evaluated by CDU between November 2022 and August 2023. Stenosis was classified as measurable or severe when > 20% or ≥ 50%, respectively. A health questionnaire was administered to assess conventional cardiovascular (CV) risk factors. Ultra-low-dose computed tomography (ULDCT) scans were analysed using an automated AI-driven CAC quantification software, with CAC expressed as the Agatston score. A retrospective analysis was performed to identify individuals potentially eligible for lipid-lowering therapy initiation by sequentially integrating CT, clinical and CDU data.

**Results:**

Overall, 122/250 (48.8%) subjects showed measurable carotid plaques, with 18 (7.2%) classified as severe plaques. 80/240 (33.3%) subjects with absent or limited CAC (A0/A1) had measurable plaques, including 10 (55.6%) of the 18 severe plaques. In the retrospective analysis, 23 subjects (23/173, 13.3%) were deemed eligible for lipid-lowering therapy based on CAC data. Among A0/A1, a further 26 individuals were reclassified as eligible: 18/150 (12%) according to conventional CV risk factors, and 8/132 (6%) based solely on CDU findings.

**Conclusion:**

A considerable proportion of LCS participants showed carotid plaques, confirming subclinical atherosclerosis even in those with absent or limited CAC. CDU, as part of an integrated strategy, may help identify individuals eligible for lipid-lowering therapy.

**Key Points:**

***Question*** *Can CDU improve CV risk assessment by detecting subclinical atherosclerosis in LCS participants with absent or limited coronary calcifications*?

***Findings*** *Measurable carotid plaques (> 20%) were detected in 33.3% participants with absent or limited coronary calcifications (A0/A1). CDU findings reclassifying 5% of A0/A1 subjects as eligible for lipid-lowering therapy*.

***Clinical relevance*** *CDU may reveal subclinical atherosclerosis in LCS participants with absent or limited coronary calcifications, improving CV risk assessment and identifying individuals who may benefit from lipid-lowering therapy initiation*.

**Graphical Abstract:**

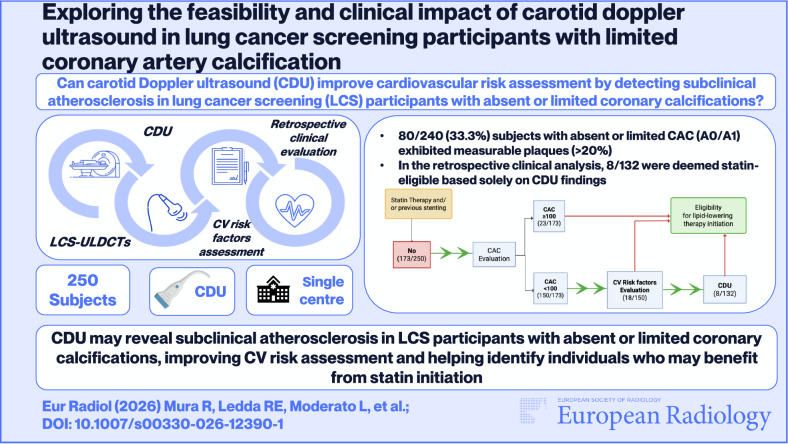

## Introduction

Cardiovascular disease (CVD) encompasses several conditions affecting the circulatory system, including coronary artery disease (CAD), heart failure, cerebrovascular disease, and peripheral vascular disease [1]. CVD displays several risk factors [2], including cigarette smoking, which is shared with lung cancer (LC) and chronic obstructive pulmonary disease [3–5].

Low-dose computed tomography (LDCT), routinely performed in lung cancer screening (LCS) programmes, allows the quantification of coronary artery calcium (CAC) [6–9], which represents a strong predictor of CAD [10]. However, the absence of CAC does not equate to a lack of CV risk; instead, these subjects fall into a *“low-risk*” rather than a “*no-risk*” group [11]. It is precisely in this subgroup of subjects—namely, those with limited or absent CAC—that complementary vascular imaging could have its greatest impact by unveiling subclinical atherosclerosis [12–14].

Carotid Doppler ultrasound (CDU) can detect calcified or non-calcified subclinical carotid plaques, and their identification can act as a CV risk stratification modifier, potentially granting lipid-lowering therapy administration [15]. Moreover, carotid plaques are associated with an increased risk for cerebrovascular events [16], which have also been reported with an incidence of approximately 0.8-1% in large LCS cohorts [17, 18]. Therefore, in a subset of subjects with either absent or limited CAC on LCS-LDCTs and without ongoing lipid-lowering therapy, implementing a CDU evaluation may provide additional insight into subclinical atherosclerosis and eventually support therapeutic decisions, which can represent a clinically meaningful endpoint in CV risk management.

We prospectively performed CDU in participants of an LCS programme, aiming to assess the prevalence of carotid plaques and their potential contribution to therapeutic recommendations regarding lipid-lowering therapy initiation—within a framework of an integrated CV evaluation—particularly in participants with absent or limited CAC.

## Materials and methods

### Study population

This prospective study included consecutive volunteers participating in the “Model for Optimised Implementation of Early Lung Cancer Detection: Prospective Evaluation Of Preventive Lung Health (PEOPLHE)” trial, a multicenter Italian LCS programme [19]. In brief, LCS-eligible participants were (a) aged 50–75 years, (b) current smokers with ≥ 15–20 pack-years, or former smokers with the same smoking history who stopped ≤ 10 years before, and (c) enroled in the coordinating Unit of the PEOPLHE trial (University Hospital of Parma). The exclusion criteria of the LCS project were (a) neoplasms within the previous five years and (b) chest CT performed in the last 12 months.

**Fig. 1:**
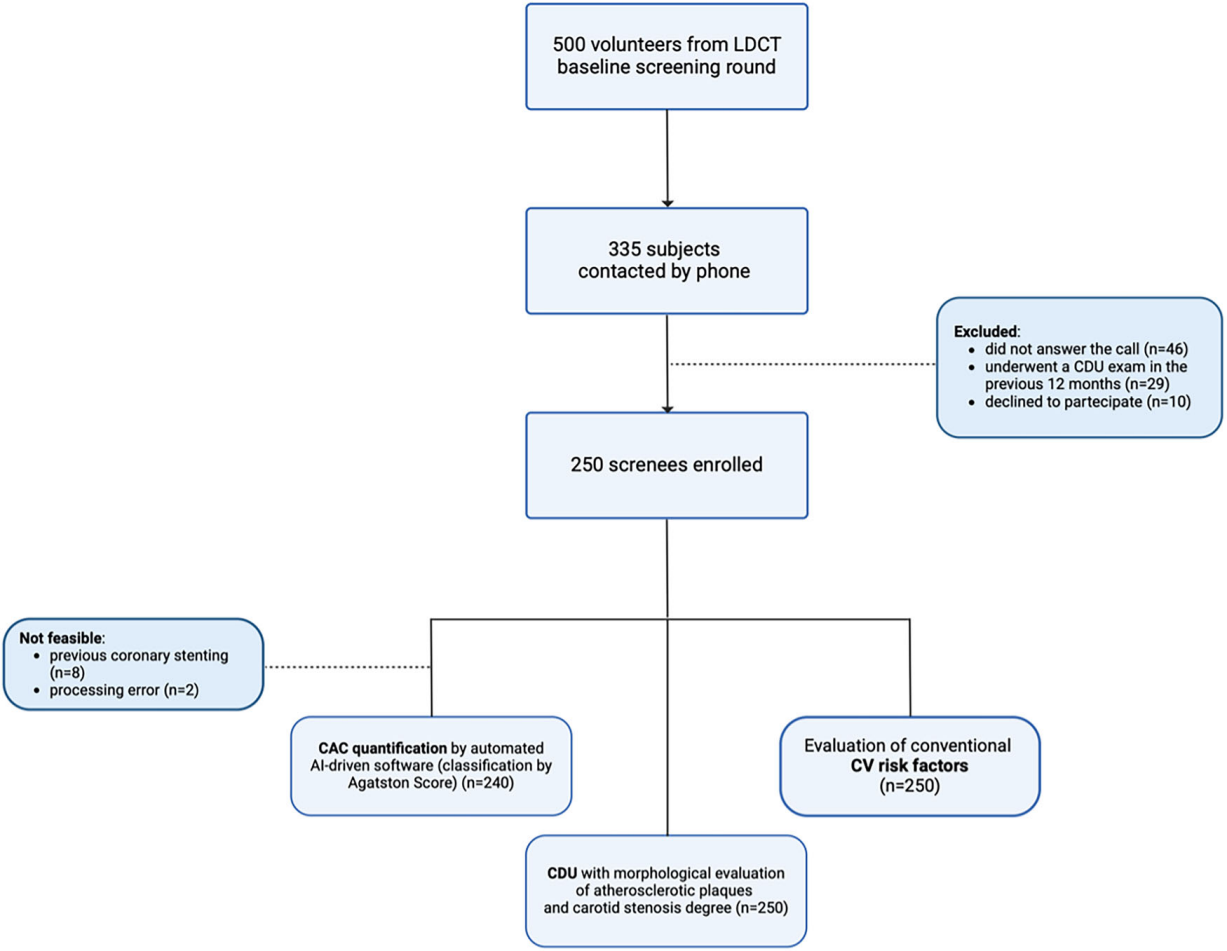
Participant flow diagram. Created in BioRender. Mura, R. (2025) https://BioRender.com/k45h306. CAC, coronary artery calcification; CDU, carotid doppler ultrasound; CV, cardiovascular; LDCT, Low-dose computed tomography

All PEOPLHE participants, except those who had undergone a CDU examination in the last 12 months, were eligible to participate in the current analysis. Participants were recruited from November 2022 to August 2023. Ethical approval was obtained from the local Ethics Committee (AVEN Ethics Committee: protocol n. 87/2022/SPER/UNIPR), and all participants provided written informed consent prior to enrolment. The patients’ selection is summarised in Fig. 1.

A resident radiologist administered a health questionnaire to identify the following CV risk factors: age, sex, smoking history, hypertension, diabetes, dyslipidemia, previous CV events and/or CV surgical intervention, family history of CV events, body mass index (BMI), and ongoing lipid-lowering treatment.

### Imaging acquisition and analysis

#### Ultra-low dose CT (ULDCT) protocol and coronary artery calcification (CAC) quantification

Chest CT for LCS was performed using an ultra-low-dose CT (ULDCT) protocol. Baseline ULDCTs were obtained on a 128-slice CT scanner (SOMATOM go.Top, Siemens Healthineers) equipped with a tin filter (Sn). Each participant was scanned in cranio-caudal direction, in one inspiratory breath-hold (details in Supplementary Materials). For CAC quantification, we used reconstruction soft kV-adjusted kernel (Sa36), slice thickness 1 mm, and increment 0.7 mm, supported by previous literature indicating that CAC scoring is more accurate with 1mm-LDCT than thicker slices [20–22].

Images were analysed using an automated artificial intelligence (AI)-driven CAC quantification software (AVIEW, Coreline Soft), which identifies areas of calcification with attenuation values above 130 Hounsfield Units (HU). The software has also been evaluated on ULDCT scans, showing good agreement with standard low-dose protocols [23]. Quantitative results were extracted from the software-generated reports by a single investigator (R.M.), and a basic visual quality check of the output was performed to exclude processing errors. CAC were reported according to the Agatston score [24], both as an absolute value in continuous scale (in Agatston units (AU)) and classified into the following four risk categories:

- A0: very low risk (AU = 0);
- A1: mild increased risk (AU = 1–99);
- A2: moderately increased risk (AU = 100–299);
- A3: moderate to severely increased risk (AU ≥ 300) [25].

#### CDU and evaluation of atherosclerotic plaques

CDU was performed using either a Philips Affiniti 70 G Ultrasound System (Philips Healthcare) or an RS85 Prestige Ultrasound System (Samsung Healthcare) with linear array transducers (3–12 MHz and 2–9 MHz, respectively), by four radiologists experienced in ultrasound imaging (M.S., G.M., R.E.L. and S.S. with 10, 8, 5, and 5 years of experience). Both longitudinal and transverse views of carotid arteries along their anatomical course and of vertebral arteries were obtained (details in Supplementary Material). These vessels were assessed in B-mode, colour, and pulsed Doppler mode for the presence of atherosclerotic plaques and degree of stenosis, as follows:

- Intima-media thickness (IMT) was measured on the distal common carotid artery (CCA) at the far wall on longitudinal scans and was considered abnormal if > 0.9 mm [26, 27].
- Plaques: structures encroaching into the vessel lumen and resulting in an IMT of > 1.5 mm or a thickness increase > 50% compared to the surrounding segment were considered plaques [27]. Plaques were characterised by number, location (CCA; external bifurcation; internal carotid artery; external carotid artery) and echogenicity, namely echo-lucent or echogenic plaque, the latter made of fibrous tissue or calcification. We calculated the degree of stenosis according to ECST criteria, and reported it at 5% intervals. Stenosis was considered measurable or severe when > 20% or ≥ 50%, respectively. The Doppler analysis of peak systolic velocity (PSV) was performed at the level of the stenosis, with a significant hemodynamic effect indicated by PSV > 125 cm/s, as part of the standard clinical assessment.

### Retrospective data analysis

A single cardiologist (L.M., 8 years of experience) performed a retrospective analysis to evaluate whether—in an LCS scenario—the availability of CDU findings could enhance CAC-based assessment in identifying individuals potentially eligible for lipid-lowering therapy. In the LCS workflow, comprehensive clinical data required for validated CV risk scores are not routinely collected. Real-world clinical decisions, like the recommendation to initiate lipid-lowering therapy, were considered effective surrogate endpoints, helping to evaluate the potential clinical utility of CDU. The analysis involved sequential integration of data from the following three domains:

- CT data: CAC expressed as Agatston score;
- Clinical data: conventional CV risk factors (age, sex, smoking history, hypertension, diabetes, dyslipidemia, previous CV events and/or CV surgical intervention, family history of CV events, BMI) and current therapies;
- CDU data: the presence of measurable and severe carotid plaques.

These domains acted as decision-making drivers to determine eligibility for lipid-lowering therapy, based on the 2024-European-ESC guidelines for the management of arterial and aortic disease and 2021-ESC Guidelines on CVD prevention in clinical practice [15, 28].

Subjects already receiving lipid-lowering therapy and/or previously treated with coronary stenting were excluded from the retrospective analysis to minimise potential confounding. In line with the routine information flow available in a real-world LCS setting, the evaluation of subjects proceeded in three sequential phases.

Calcium score data were assessed first. According to the 2019-ESC/EAS Guidelines, a CAC score > 100 AU can act as a CV risk modifier [29]; therefore, participants with AU ≥ 100 (A2–A3 categories) were classified as high-risk and deemed eligible for lipid-lowering therapy, independently of their clinical risk factors or CDU findings [29, 30]. Second, in individuals with a CAC score ranging from 0 to 99 AU (A0–A1 categories), the need for lipid-lowering therapy initiation was evaluated based on conventional CV risk factors. Finally, CDU data were integrated to explore its potential role as a therapeutic decision-support tool in those subjects for whom CAC and clinical data would not have otherwise suggested its initiation.

### Statistical analysis

The Kruskal–Wallis test was used to compare categorical and continuous features, whereas the chi-square test was used to perform univariate comparisons between categorical variables; when the assumptions of the chi-square test were not met, Fisher’s exact test was applied instead. Based on the data prospectively collected for clinical, CAC and CDU data, we retrospectively evaluated the potential role of CDU as a further criterion beyond clinical and LDCT parameters to determine eligibility for lipid-lowering therapy. A McNemar test was used to assess whether the addition of CDU to the baseline evaluation model (CAC score and conventional CV risk factors) significantly changed.

Analyses were performed with R (version 4.3.1; R Core Team, 2023) [31] and Jamovi Statistical Software Version 2.6. For all analyses, a *p*-value < 0.05 was deemed statistically significant.

## RESULTS

### Population characteristics

The study population included 250 subjects who underwent CDU: 132 males (52.8%), with a median age of 62 years (IQR:57-67). Current smokers were 194/250 (77.6%), with a median pack-years of 30 (IQR:24-40).

A sizeable proportion of the study participants was either overweight (113/250, 45.2%) or obese (28/250, 11.2%). A history of dyslipidemia was reported in 116/250 (46.4%). Previous CV events were reported in 13/250 participants (5.2%), of whom 2/13 (15.4%) had a transient ischaemic attack and 1/13 (7.7%) had a stroke. The characteristics of participants are summarised in Table 1.

**Table 1:** Characteristics of study participants

Characteristics of subjects	Subjects *n* = 250 (%)
Age (y)	
Median [IQR]	62 [57–67]
Sex	
Male	132 (52.8)
Female	118 (47.2)
Smoking history	
Current smoker	194 (77.6)
Former smoker	56 (22.4)
Pack-years (median number, IQR)	30 [24–40]
CV risk factors	
Hypertension	97 (38.8)
Dyslipidemia	116 (46.4)
Diabetes	15 (6)
Family history of CV events	131 (52.4)
Previous CV events	13 (5.2)
Ischaemic heart disease	9
Stroke	1
Pericarditis	1
Transient ischaemic attack (TIA)	2
Previous surgical CV intervention	
Carotid endarterectomy (CEA)	1 (0.4)
Percutaneous coronary intervention (PCI)	8 (3.2)
Treatment with statins	74 (29.6)
BMI	
< 18.5	8 (3.2)
18.5–24.9	101 (40.4)
25–29.9	113 (45.2)
> 30	28 (11.2)

Categorical variables were presented as count (proportion), and continuous variables were presented as median (IQR)

### CAC and CDU assessments

#### CACs

240 out of 250 (96%) ULDCTs were processed by the automated AI-driven CAC quantification software: CAC quantification was not feasible in 10 (4%) due to previous coronary stenting (8/10, 80%) or processing error (2/10, 20%). Frequencies of individual CAC categories were as follows: 64/250 (25.6%) A0, 131/250 (52.4%) A1, 23/250 (9.2%) A2, and 22/250 (8.8%) A3. Among the coronary arteries, the left anterior descending (LAD) was the most frequently involved (LAD 30%; right coronary 24%; left main 23%; left circumflex 23%).

Most CV risk factors were more frequently reported in subjects with higher CAC (Table 2).

**Table 2:** Association between conventional CV risk factors and coronary artery calcium, classified according to Agatston score categories

	Coronary artery calcium score (AGATSTON SCORE)				*p*-value
	A0	A1	A2	A3	
Subjects [*n* (%)]	64 (26)	131 (52)	23 (9)	22 (9)	
Sex					
Male	21 (8)	73 (29)	14 (6)	16 (6)	**0.002**
Female	43 (17)	58 (23)	9 (4)	6 (2)	
Age, y (Median, IQR)	59 [56–64]	61 [56–65]	65 [59.5-68.5]	68 [64.25-71]	**< 0.001**
Current smoker^*^	48 (19)	108 (43)	19 (8)	14 (6)	0.199
Dyslipidemia	21 (8)	61 (24)	15 (6)	13 (5)	**0.024**
Hypertension	14 (6)	53 (21)	11 (4)	12 (5)	**0.012**
Diabetes^*^	0 (0)	8 (3)	3 (1)	3 (1)	**0.011**
Previous CV events^*^	0 (0)	3 (1)	2 (1)	3 (1)	**0.008**
Family history	28 (11)	77 (31)	6 (2)	12 (5)	**0.016**
BMI^*^					
< 24.9	42 (17)	51 (20)	7 (3)	7 (3)	**0.001**
25–29.9	19 (6)	62 (25)	10 (4)	14 (6)	
> 30	3 (1)	18 (7)	6 (2)	1 (0)	

*p*-values were derived from the Kruskal–Wallis test for “Age, y”, from the chi-square test for categorical variables, and from Fisher’s exact test for categorical variables marked with (*). Percentages are calculated using the total study population as the denominator

Bold values indicate statistical significance *p* < 0.05

*CV* cardiovascular

#### CDU

Increased IMT was reported in 54/250 subjects (21.6%). Carotid plaques were detected in 162/250 (64.8%) subjects; measurable plaques were found in 122 (48.8%) subjects, of whom 18 (7.2%) had severe plaques. 114 (93.4%) out of the 122 measurable plaques were echogenic, whereas 8/122 (6.6%) were echo-lucent.

Among the 122 subjects with carotid plaques, 114 (93.4%) CT scans could be processed by AI-driven software for CAC scoring: 18/114 (16%) A0, 62/114 (54%) A1, 15/114 (13%) A2, and 19/114 (17%) A3 (Table 3). In particular, no severe plaques were observed in A0, whereas detected in 10/62 (16%) A1, 1/15 (6.7%) A2, and 5/19 (26%) A3. Amongst 8/122 subjects who showed echo-lucent plaques, 4/8 (50%), 2/8 (25%), and 1/8 (12.5%) were respectively classified as categories A0, A1, and A3, thus representing a sizeable proportion of at-risk subjects (6/8, 75%) not identified by CT; in 1/8 (12.5%) subjects with echo-lucent plaques, CAC was not feasible for processing error.

**Table 3:** Distribution of carotid plaques across Coronary Artery Calcium Score (Agatston score) categories

CDU data	Total subjects [*n* (%)]	Coronary Artery Calcium Score (AGATSTON SCORE) Group			
		A0 [*n* (%)]	A1 [*n* (%)]	A2 [*n* (%)]	A3 [*n* (%)]
No measurable plaques	126 [50.4]^*^	46 [36%]	69 [54%]	8 [6%]	3 [2%]
Total measurable plaques (> 20%)	114 [45.6]^**^	18 [15%]	62 [51%]	15 [12%]	19 [16%]
“High grade” (≥ 50%) subgroup^#^	16/114 [14%]^***^	0	10 [8.8%]	1 [0.9%]	5 [4.4%]

^*^ Two subjects are missing because the corresponding CT scans were not processed by AI software due to previous coronary stenting

^**^ Eight subjects are missing because the corresponding CT scans were not processed by AI software (six for previous coronary stenting, two due to processing error)

^***^ Two subjects are missing because the corresponding CT scans were not processed by AI software due to previous stenting

^#^ Severe plaques (≥ 50%) are a subset of total measurable plaques (> 20%), as detailed in the table

*CDU* carotid doppler ultrasound

The remaining 126/240 (52.5%) plaque-free subjects were more frequently included in lower CAC groups: A0 in 46 (36%), A1 in 69 (54%), A2 in 8 (6%), and A3 in 3 (2%) (*p* < 0.001).

PSV were reported descriptively according to the degree of stenosis (21–49% and ≥ 50%). Median PSV was 60 cm/s (IQR 50–80 cm/s) in the 21–49% group and 75.5 cm/s (IQR 55–101 cm/s) in the ≥ 50% group. In the ≥ 50% group, PSV > 125 cm/s was found in two subjects (135 and 165 cm/s, respectively).

### Retrospective evaluation of eligibility for lipid-lowering therapy

Of the 250 participants, 173 (69.2%) were included in the retrospective clinical evaluation. A total of 74/250 subjects were excluded due to ongoing for lipid lowering therapy, and an additional 3/250 due to a history of coronary stenting.

Following retrospective cardiological assessment, 49 out of the 173 lipid-lowering treatment-naïve subjects (28.3%) were deemed eligible for such treatment. Specifically, 23/173 (13.3%) showed CAC score A2/A3, and therefore were considered eligible in accordance with current ESC/EAS recommendations [28, 29]. Of these, 19/23 (82.6%) also exhibited measurable plaques on CDU, with 3/19 (15.8%) classified as severe.

**Fig. 2:**
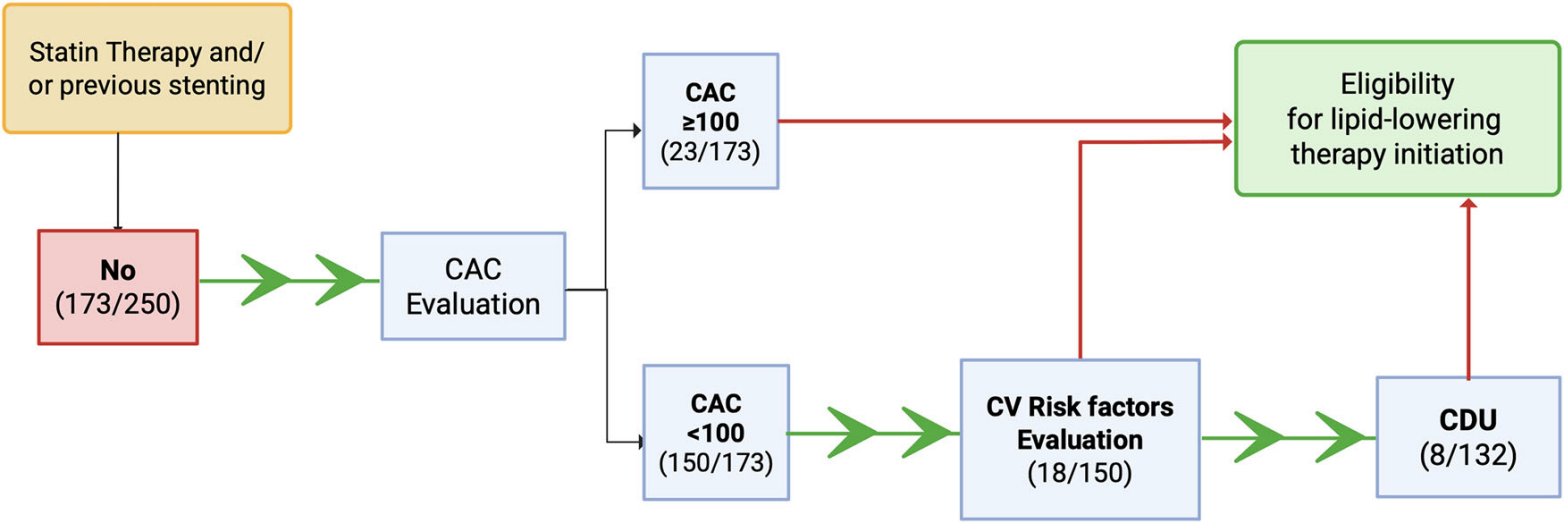
Flowchart illustrating the sequential integration of CAC, clinical risk factors, and CDU findings to determine eligibility for lipid-lowering therapy in LCS participants. CAC, coronary artery calcification; CV, cardiovascular; CDU, carotid Doppler ultrasound; LCS, lung cancer screening. Created in BioRender. Mura, R. (2025) https://BioRender.com/xyxaq8m

Among the remaining 150/173 (86.7%) categorised as A0/A1, 18/150 (12%) were considered as eligible for lipid-lowering therapy based on conventional CV risk factors. In this group, measurable carotid plaques were present in all 18 subjects (100%), although none were classified as severe. Finally, 8/132 participants (6%) were considered eligible for lipid-lowering therapy based solely on CDU findings (Fig. 2). Notably, 7 of these 8 individuals (87.5%) exhibited severe plaques, while all of them were included in the A1 category, with a median Agatston score of 23 AU [IQR:13.25-49.25]. There was a difference in lipid-lowering treatment eligibility when CDU findings were included, compared with clinical and CT data alone (*p* = 0.005, Table 4).

**Table 4:** The McNemar test shows a difference in statin eligibility when CDU findings were included, compared with clinical and CT data alone

Contingency table			
	Clinical and CT and CDU		
Clinical and CT data	Statin: no	Statin: yes	Total
Statin: no	124	8	132
Statin: yes	0	41	41
Total	124	49	173

McNemar *p* = 0.005

*CDU* carotid doppler ultrasound

## Discussion

Based on the results of this prospective study conducted within an LCS setting, we observed that carotid plaques were frequently detected, with a prevalence of 49% (122/250) in the overall cohort. As expected, the burden of carotid plaques increased with higher CAC scores; however, we also confirmed the presence of subclinical atherosclerosis in participants with absent or limited CAC. Specifically, measurable carotid plaques were identified in one-third of subjects (80/240, 33.3%) with either no or limited CAC (18/240 A0, 62/240 A1), and 10 out of the 18 (55.6%) subjects with severe plaques were classified as A1. Unsurprisingly, approximately 75% of individuals categorised as A2/A3 exhibited measurable carotid plaques. Consistent with previous studies, these findings underscore the jeopardised distribution of subclinical atherosclerosis, especially among heavy smokers [14, 32, 33]. Moreover, of the eight subjects with echo-lucent plaques, 4 (50%) were classified as A0 and 2 (25%) as A1. Identifying these so-called “unstable plaques” can be clinically meaningful, as they are more frequently associated with an increased risk of acute ischaemic cerebrovascular events [34].

Our study findings highlight that CDU may provide additional value for identifying subclinical atherosclerosis even in apparently low-risk subjects such as those with no or limited CAC (categories A0/A1). Conversely, in individuals classified as A2/A3, further evaluation with CDU is unlikely to significantly impact treatment decisions or prognosis, as these are already considered at-risk [29, 30]. Indeed, CAC has been extensively investigated in the context of LCS, and the presence of moderate to severe CAC (categories A2/A3) has already been associated with both fatal and non-fatal CAD events [35–37]. Consistent with previous studies, the prevalence of CAC in our study’s cohort was 70.4% [38, 39]. Moreover, recent studies have further underscored the role of CAC in CV risk stratification, showing that its presence improves risk prediction even among individuals classified as low-to-borderline risk based on clinical scores alone [40]. However, the assumption that a calcium score of zero confers no CV risk might not be entirely accurate [41, 42]. In the LCS-eligible MESA cohort, 20.8% of subjects with an intermediate risk clinical score and no CAC experienced at least one CV event (including strokes and both fatal and non-fatal CAD events) after a mean of 11.1 years of follow-up [41, 43]. Notably, the 10-year CV event rate for this subgroup was nearly three times higher than that of the entire MESA cohort, highlighting the potentially significant impact of implementing primary prevention in LCS participants [44]. Moreover, previous studies assessing CV risk in LCS cohorts showed that a substantial proportion of individuals eligible for lipid-lowering therapy, or classified as intermediate risk based on clinical criteria, exhibited no detectable CAC [41, 44]. Thus, integrating various imaging modalities could enhance CV risk stratification, potentially prompting earlier initiation of lipid-lowering therapy.

According to Mehta et al, the heterogeneity in the long-term CV risk observed in individuals with absent CAC may be directly related to subclinical carotid atherosclerosis [11], and, as outlined by the 2021-ESC Guidelines, a more comprehensive atherosclerosis assessment by CDU can act as a risk modifier [15]. Based on these assumptions, we expanded our study to retrospectively investigate whether the systematic implementation of CDU within the LCS pathway could provide additional information to guide preventive treatment strategies beyond what can be derived from conventional CAC scoring. To the best of our knowledge, this is the first study to explore the complementary role of CDU in this setting.

Previous studies reported that up to 80% of LC-screnees who are eligible for lipid-lowering treatment were not receiving therapy, underscoring the potential of LCS programmes to serve as a broader “safety net”[43–45]. Similarly, nearly 70% of the study participants were not under treatment with lipid-lowering therapy. Based on CAC data, 23 subjects in A2/A3 groups (23/173, 13.3%) were eligible for starting lipid-lowering therapy, while, among A0/A1, a further 26 individuals (17.3%) were deemed eligible for lipid-lowering therapy based on CV risk factors (18/150, 12%) and CDU findings (8/132, 6%), respectively. These eight individuals, all classified as A1 (median 23 AU) and lacking significant conventional CV risk factors, were reclassified as eligible for lipid-lowering therapy solely due to CDU findings.

Although the proportion of individuals reclassified based on CDU was relatively modest (5%, i.e. 8/150), this finding may become clinically relevant when applied to larger populations. In the context of primary prevention, the number needed to treat (NNT) with statins to prevent one major CV event over 10 years ranges between 19 and 28 [46, 47]. This implies that even small increases in detection could yield significant clinical benefits. For instance, in the bioMILD trial, only 22% of more than 3000 individuals not receiving statins had CAC scores ≥ 100. If a similar 5% rate of CDU-based reclassification were applied to such a cohort, approximately 150 additional high-risk individuals could be identified. Based on an average NNT of 19–28, treating these patients could potentially prevent 5–8 major CV events, which can be regarded as clinically meaningful.

The global burden of CVD continues to rise, not only in terms of deaths and disability-adjusted life years, but also in costs, including healthcare and indirect costs, such as those related to lost economic productivity due to morbidity and premature mortality [48]. Participating in an LCS programme can represent a teachable moment to promote CVD prevention through lifestyle changes and initiation of primary prevention therapies [49]. This has been well-demonstrated, for example, by a sub-study from the ROBINSCA trial, which highlights how receiving a CT screening result increased participant prevention-seeking behaviour compared with traditional risk assessment [45, 49]. Building on this concept, adherence to our initiative was rather high, as only 10/335 (3%) subjects initially contacted declined the invitation.

However, while CDU remains a cost-effective, non-invasive, and radiation-free technique, its systematic implementation in all individuals classified as A0/A1 may raise concerns about feasibility. In our cohort, 150 CDU examinations were required to identify eight individuals with carotid plaques that would independently justify the initiation of a lipid-lowering therapy. This finding highlights both the potential value and the inherent limitations of CDU in the LCS scenario. Thus, although potentially reasonable from a clinical perspective, future strategies might benefit from a more targeted approach to optimise resource utilisation. In this light, optimising patient selection becomes a key challenge for future and sustainable applications of multidimensional approaches for improving CV risk stratification. The potential clinical impact of CDU seems most significant in participants with low or absent CAC in whom CT findings alone may underestimate subclinical atherosclerosis. Within this subgroup, adding specific imaging characteristics—such as emphysematous changes or aortic calcification—might help refine selection for additional US evaluation. Although our study was not designed to identify such predictors (as we prospectively performed CDU without preliminary evaluation of other imaging descriptors), exploring these correlates could be a valuable direction for future research, supporting a tailored approach to CV risk assessment in LCS populations.

This study has some limitations. First, it is a single-centre study with a relatively small population. Second, conventional CV risk factors were self-reported through a health questionnaire. In particular, we observed some inconsistencies as three subjects reported a history of coronary stenting but no lipid-lowering therapy. Furthermore, due to the relatively short follow-up, information on subsequent CV events was not collected, and further cost-effectiveness analyses were not performed, as they were beyond the scope of the present study.

In conclusion, our study results reveal a sizeable proportion of subjects with carotid plaques in participants of an LCS programme, confirming the presence of subclinical atherosclerosis even in individuals with absent or limited CAC.

## Supplementary information

Supplementary information

## Abbreviations

AU: Agatston unit
AI: Artificial intelligence
BMI: Body mass index
CV: Cardiovascular
CVD: Cardiovascular disease
CDU: Carotid Doppler ultrasound
CCA: Common carotid artery
CAC: Coronary artery calcification
CAD: Coronary artery disease
HU: Hounsfield unit
IMT: Intima-media thickness
LAD: Left anterior descending
LDCT: Low-dose computed tomography
LCS: Lung cancer screening
NNT: Number needed to treat
ULDCT: Ultra-low dose CT
