# Supplementary material for: Exploring the prevalence and clinical impact of carotid plaque burden by Doppler ultrasound in lung cancer screening participants with limited coronary artery calcification
Source: Eur Radiol. 2026 Feb 24;36(7):5482–91. doi: 10.1007/s00330-026-12390-1 (PMC13282308; doi:10.1007/s00330-026-12390-1)
Supplement: Supplementary file 1 — Supplementary information [file 330_2026_12390_MOESM1_ESM.pdf]

Exploring the Prevalence and Clinical Impact of Carotid Plaque Burden by Doppler Ultrasound in Lung Cancer Screening Participants with Limited Coronary Artery Calcification

ELECTRONIC SUPPLEMENTARY MATERIAL

CT Scanning Parameters

All ULDCT scans were obtained on a 128-slice CT scanner (SOMATOM go.Top, Siemens Healthineers) equipped with a tin filter (Sn), with the following parameters: fixed tube-voltage, 100kVp with tin filter; automated exposure control (AEC) for current at reference 100 mAs; collimation, 0.625 mm; pitch, 0.8; rotation time, 0.33 seconds.

Carotid Doppler ultrasound

Carotid Doppler ultrasound (CDU) was performed using either a Philips Affiniti 70G Ultrasound System (Philips Healthcare) or an RS85 Prestige Ultrasound System (Samsung Healthcare) with linear array transducers (3-12 MHz and 2-9 MHz, respectively). Four radiologists experienced in ultrasound imaging (MS, GM, REL and SS with 10, 8, 5, and 5 years of experience) conducted all examinations following a standardized scanning approach. Both longitudinal and transverse views of the carotid arteries along their anatomical course - from the common to the internal and external carotid arteries, as distally as possible - and of the vertebral arteries were obtained.
